# Vegetation Pattern Modulates Ground Arthropod Diversity in Semi-Arid Mediterranean Steppes

**DOI:** 10.3390/insects11010059

**Published:** 2020-01-18

**Authors:** Fernando Meloni, Berta F. Civieta, Juan A. Zaragoza, María Lourdes Moraza, Susana Bautista

**Affiliations:** 1 Departamento de Física, Faculdade de Filosofia, Ciências e Letras de Ribeirão Preto, Universidade de São Paulo, CEP 14040-901 Ribeirão Preto, Brazil; 2 Department of Ecology and IMEM, University of Alicante, E-03690 San Vicente del Raspeig, Alicante, Spain; bertac43@gmail.com (B.F.C.); juan.zaragoza.miralles@gmail.com (J.A.Z.); s.bautista@ua.es (S.B.); 3 National Institute of Science and Technology for Complex Systems, CEP 22290-180 Rio de Janeiro, Brazil; 4 Department of Environmental Biology, University of Navarra, E-31080 Pamplona, Spain; mlmoraza@unav.es

**Keywords:** arthropods diversity, drylands, soil fauna, spatial pattern, vegetation patches

## Abstract

The ecological functioning of dryland ecosystems is closely related to the spatial pattern of the vegetation, which is typically structured in patches. Ground arthropods mediate key soil functions and ecological processes, yet little is known about the influence of dryland vegetation pattern on their abundance and diversity. Here, we investigate how patch size and cover, and distance between patches relate to the abundance and diversity of meso-and microarthropods in semi-arid steppes. We found that species richness and abundance of ground arthropods exponentially increase with vegetation cover, patch size, and patch closeness. The communities under vegetation patches mainly respond to patch size, while the communities in the bare-soil interpatches are mostly controlled by the average distance between patches, independently of the concurrent changes in vegetation cover. Large patches seem to play a critical role as reserve and source of ground arthropod diversity. Our results suggest that decreasing vegetation cover and/or changes in vegetation pattern towards small and over-dispersed vegetation patches can fast lead to a significant loss of ground arthropods diversity in drylands.

## 1. Introduction

Drylands are arid, semiarid, and dry-subhumid ecosystems [1] that typically exhibit spatially patterned vegetation, with plants grouped in patches in a matrix of exposed soil. Vegetation patchiness determines most soil processes and functions in drylands [2,3,4,5,6,7] and offers heterogeneous conditions for soil fauna to develop [8,9]. Dryland degradation (e.g., due to disturbances such as grazing, fire, and drought) commonly reduces the vegetation cover and alters the spatial pattern of vegetation [10,11,12]. These changes can, in turn, reduce water infiltration and increase soil erosion, thereby leading to further degradation [3,13,14], and potentially triggering abrupt changes in the ecosystems towards degraded stable states [15,16,17]. Several studies have pointed to the consequences of decreasing vegetation cover on the physical, chemical, and microbiological components and properties of dryland soils [16]. However, soil fauna, such as ground arthropods, have received little attention in the context of dryland degradation [9,18].

We define “ground arthropods” in a broad sense as all arthropods that spend their entire or partial lifetime in the litter or soil [19]. This definition includes the euedaphic (true edaphic), epiedaphic (surface explorers), and hemiedaphic (scavengers that live in galleries) groups [20]. Ground arthropods are rather ubiquitous in nature, occurring even under the most arid conditions [21,22]. Directly or indirectly, the various groups of ground arthropods play distinct roles in ecosystem engineering, dispersion of microorganisms, and population regulation, affecting water infiltration, decomposition, and nutrient cycling, among other soil processes [23,24,25,26,27]. Consequently, changes in species composition or community structure of ground arthropods may affect key soil ecological functions and services [28].

Experimental studies demonstrated that changes in plant communities along successional dynamics relate to the diversity of ground-arthropod communities [9,29], a relationship often reported in the context of bioindication [30,31,32]. Hence, we presume that changes in the vegetation structure (cover and spatial pattern) of dryland ecosystems could also be linked to changes in ground-arthropod communities, especially in the distribution of meso- and microarthropods. Empirical evidence shows that environmental filters in drylands are essential in explaining where macroarthropods (>1 cm) live, suggesting that plants provide protection, habitat, and resources for them [21,33,34,35,36,37]. However, less is known about smaller arthropods and, concerning arthropods, body size definitively matters [27,38,39]. Macroarthropods can build or hollow out shelters in litter and soil, move through the landscape for considerable distances and actively search for resources. Conversely, the meso-and microarthropods (<1 cm) show limited mobility on the soil surface, often use already existing shelters and are more vulnerable to sunlight and desiccation effects. Therefore, the distribution of meso-and microarthropods may be inferred to be strongly linked to the pattern of vegetation patches. Specifically, it could be hypothesized that the spatial pattern of vegetation controls the diversity of ground meso-and microarthropods, affecting the probability of viable populations to establish, grow and migrate. If so, the degradation of the vegetation spatial structure could lead to the loss of particular functional groups of arthropods, which in turn could largely impair soil functioning.

In this paper, we investigate how variations in the spatial pattern of dryland vegetation affect the abundance, diversity, composition, and spatial distribution of ground meso-and microarthropods. Taking the semi-arid Mediterranean steppes of southern Spain as a dryland model, we aimed to determine how vegetation cover, patch size, and between-patch distance influence the abundance, richness, and high-level taxa composition of species of ground arthropods. The three landscape metrics selected for this study have proven to be critical control factors of dryland biotic structure and functioning. Thus, both vegetation cover and patch size largely explain soil and ecosystem multifunctionaliy [40,41], and plant species richness [42], while key soil functions such as water infiltration and nutrient cycling largely depends on the extent, connectivity and functioning of bare-soil interpatches [43,44,45]. Based on these previous findings, we hypothesized that increasing vegetation cover and/or patch size would increase ground arthropod abundance and species richness, while increasing distance between vegetation patches would independently exert the opposite effect. We assessed the relationships between vegetation and ground meso-and microarthropods variables at two scales: landscape-based level (sampling plots with mosaics of patches and interpatches) and sample-based level (individual patches and interpatch areas). We also investigated whether the responses of ground arthropods are consistent between patch and interpatch microhabitats, and between the litter and the soil layers. Our results show a strong dependence of the abundance and diversity of ground arthropods on the spatial pattern of dryland vegetation, which may largely contribute to reinforce the pattern-function link of dryland landscapes.

## 2. Methods

### 2.1. Study Area

This study was carried out in Cabezo de la Plata, Murcia, Spain (UTM 23 S, coordinates 673831 E to 675919.78 E; 42030440 N to 42011716 N). The climate is semiarid (~300 mm/year of precipitation and a mean annual temperature of 19 °C), with a long dry period in summer. Soils are haplic calcisols and lithic leptosols [46]. The area has been modified by human activities over millennia and is considered to be at risk of desertification [47]. The current landscape is a mosaic of abandoned agricultural land and pastures, pine afforestations, and natural semi-arid steppe. We conducted the study on semi-arid steppe areas, which are mostly located on gentle slopes, and where the most abundant plant species is the tussock grass *Stipa tenacissima* L. A general overview of the vegetation and landscape is depicted in Supplementary materials (Figure S1).

We reduced the geographic and seasonal influences on the species composition and abundance of ground fauna by minimizing the length of the sampling period to few months (July–August) and by limiting the study to an area of approximately 1 km^2^. Within this area, we selected 14 (~400 m^2^) sampling plots distributed on steppe hillslopes. The plots largely varied in vegetation cover, from barely vegetated to almost fully covered plots, as result from previous grazing and human activities. On each sampling plot, we arbitrarily selected five vegetation patches (sampling patch). The patches were mainly consisted of *S. tenacissima* tussocks, often associated to few individuals of other grass and sub-shrub species. We also defined five bare-soil sampling points (sampling interpatch), each of them in the centroid of the positions of the nearest neighboring patches. Details of the sampling design on each plot are depicted in Supplementary materials (Figure S2).

### 2.2. Vegetation Data

We characterized the spatial structure of the vegetation by measuring vegetation patch cover, patch size, and the distance between neighbor patches. Data of patch size and distance between patches were obtained from field measurements. On each sampling plot and for five sampling patches, we measured the patch diameter (cm) and the distance (cm) to each of the four nearest neighboring patches. From these measurements, we estimated three metrics at the sample-based scale: (1) *P_S(i)_*, the size (diameter) of the individual sampling patch; (2) *D_P-N_*, the average distance between the sampling patch and the four nearest patch neighbors; and (3) *D_I-N_*, the average distance between the sampling interpatch and its four nearest patches. For the landscape-based approach, we estimated the average size (*Ps*) of the sampled patches in the plot, and the average patch closeness (*Cl*), calculated as the inverse of the plot average of all the *D_P-N_* values (cm^−1^).

We calculated the vegetation cover (*V_C_*) values for each plot from digital aerial images (Google Earth^TM^ 2015). We identified and delimited the sampling plots on the aerial images and calculated their vegetation coverage using the ImageJ software [48]. We used a color threshold filtering to separate vegetation patches from the bare-soil surroundings. The threshold values were manually adjusted for each image, with most of the values set to approximately 90 for brightness and the other settings kept fixed: threshold color “Red”; color space = “HSB”; hue = “0; 255”; saturation = “0; 255” [49,50]. Next, the edited images were transformed into binary images, where black pixels represented vegetation and white pixels represented the interpatches. A patch was identified as a set of connected black pixels completely surrounded by white pixels. Subsequently, the filtered images were compared with the original ones by overlapping the layers. When necessary, small corrections were applied manually. Finally, the binary images were analyzed by ImageJ (tool box) [48], which determined the patch areas and the relative cover of vegetation patches on each plot. 

### 2.3. Arthropods Data

The arthropods were sampled from the sampling patches and interpatches on each plot. We delimited a quadrat (0.25 × 0.25 m) at each sampling point to collect all litter inside the quadrat and to take 1 L of soil (0–10 cm depth), from which the fauna was extracted. In the patch samples, a litter layer was always present, generating two distinct samples: the litter and the soil samples, which were separately collected and analyzed. For the interpatch samples, the litter layer was never present, and only the sample of the 0–10 cm soil layer was used. Each faunal sample (litter or soil) was individually bagged in the field and transported to the laboratory, being maintained in modified Berlese-Tülgren funnels for six days for faunal extraction. All collected arthropods were preserved in an ethanol solution (80%). As expected, this methodology predominantly sampled the meso-and microarthropods in comparison to macroarthropods, which were rarely collected. Next, we separated, counted and photographed the organisms using a stereo-microscope (Optech LFZ) coupled to a high-resolution digital camera (Nikon D3200) and a Leica microscope (ICC50).

Arthropod identification was achieved at different taxonomic levels. However, given the difficulties to identify a number of species, we also used morphospecies sorting as a surrogate for the taxonomic sorting at the species level, which is considered to be a sufficiently reliable approach to assessing the ecology of soil communities [19,51,52,53]. When considering the morphospecies approach, we followed a recursive and exhaustive process of comparison among specimens to identify and discard double entries, guaranteeing that each morphospecies represented only one species. For support, we used specialized literature, a large photographic bank of specimens, our own database, and expert opinions, considering all morphological variations intrinsic to sexual dimorphism, developmental stages, etc. A list of the identified taxa and literature used are presented in Supplementary materials (Table S1). Hereafter, we refer to the number of morphospecies as the number of species.

Arthropod abundance (number of individuals; *A*) and number of species, (species richness; *R*) were calculated for single patch and interpatch samples and for pooled samples for each plot and microhabitat: interpatch, patch, patch litter and patch soil. For the sample-based analysis, we used the abundance and richness values for each individual patch and interpatch sample; for the landscape-based analyses, we used the pooled-sample data. The two analytical scales provide complementary information to elucidating how the resources provided by the vegetation patches and the relative isolation imposed by the bared soil areas jointly drive the abundance and diversity of dryland soil arthropods.

### 2.4. Data Analyses

Landscape-scale effect of vegetation pattern on soil arthropod abundance and richness was assessed using ANCOVAs, with either microsite type (patches vs. interpatches) or soil layer (litter vs. soil) as fixed factor and the vegetation pattern descriptor (either patch cover, average patch size or patch closeness) as covariable. We also estimated the coefficient of determination (R^2^) of the linear regressions between each covariable and the abundance and richness of soil arthropods. Given the expected correlation between the various vegetation pattern descriptors, we additionally used partial correlation analysis to test the independent effect of each vegetation descriptor on soil fauna once the influence of other vegetation pattern descriptors is excluded.

We evaluated whether vegetation pattern affected the composition of high-level taxa by using Canonical Correspondence Analysis (CCA) [54]. To control for multicollinearity, we calculated the variance inflation factor (VIF), and no constraint was considered fully redundant (VIF < 10). The variables Vc, Ps, and Cl were used to construct the constraining matrix, while the quantities of each high-level taxa in the 14 sampling plots constituted the response matrix. Rare taxa (occurrence at only one site) were not considered for constructing the response matrix. All the statistical analyses were performed in R [55], using the vegan package [56] for the CCA.

For the sample-based analyses, we also used partial correlations to test the individual importance of each sample-level vegetation descriptor as explanatory variable for ground arthropod abundance and richness. Additionally, we graphically explored the spatial relationship between ground arthropod abundance and richness and the patch-interpatch landscape structure. To do so, we constructed a new variable composed by the average distance of the sampling interpatch to the nearest patches (*D_I-N_*) and the individual patch size *P_S(i)_*. We plotted individual interpatch and patch ground arthropod data against this new single variable, with the zero value on the *x*-axis figuratively representing the interface between interpatches and patches.

## 3. Results

We found that vegetation cover ranged from 2.2 to 85.7%, average patch size ranged from 46.9 cm to 195.0 cm, the average distance between patches varied from 69.2 cm to 469.0 cm, and patch closeness ranged from 0.0021 cm^−1^ to 0.0144 cm^−1^ over the 14 sampling plots. Altogether, the 140 sampling points resulted in a total collection of 3740 arthropods (427 individuals/m^2^), mostly concentrated in patch samples, and consisting of 255 different morpho-species. The most important groups were Acari (3098 individuals; 111 species), Collembola (129 individuals; 14 species), Psocodea (120 individuals; 2 species), Coleoptera (84 individuals; 24 species) and Araneae (59 individuals; 26 species; Figure S3 in Supplementary materials). The abundance of the ground arthropod sampled ranged from 9 to 137 individuals per patch sampling point (average = 34.1) and from 0 to 58 (average = 5.9) per interpatch sampling point. Average arthropod richness was 9.8 and 2.5 per patch and interpatch sampling points, respectively. The interpatches showed no species that were not present in the patch microhabitat.

### 3.1. Landscape-Based Analyses

**Figure 1:**
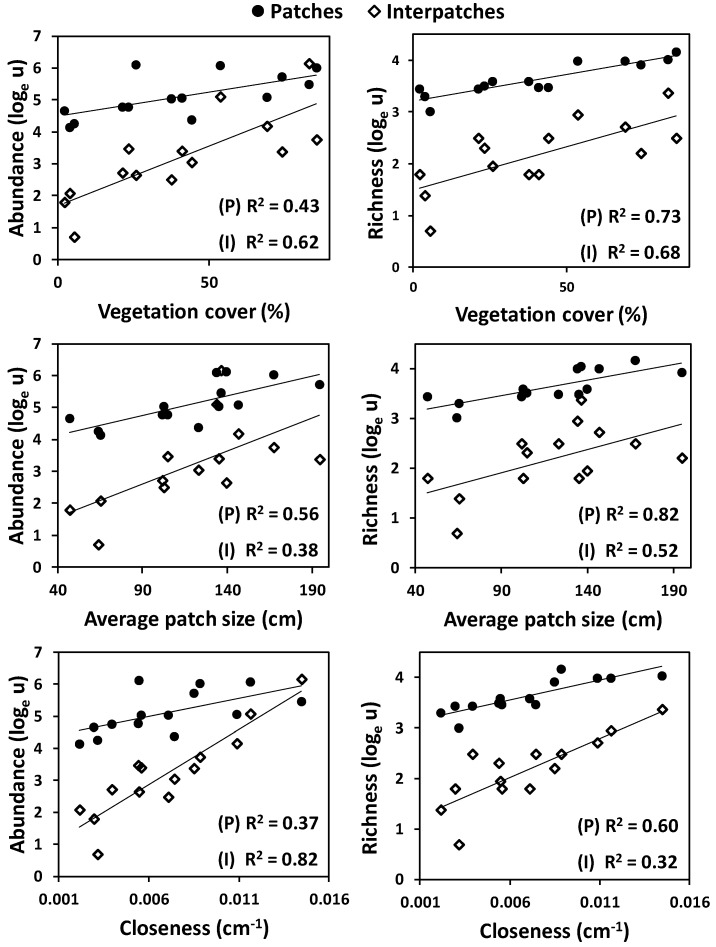
Relationships between log-scaled ground arthropod abundance (left panels) and richness (right panels) and landscape vegetation pattern metrics (vegetation cover, average patch size, and patch closeness) as a function of the type of microhabitat: Patches (P) and Inter-patches (I). Continuous lines represent statistically significant linear regressions (*p* ≤ 0.05) between the respective log-transformed response variable and the explanatory variable for each microhabitat type.

**Figure 2:**
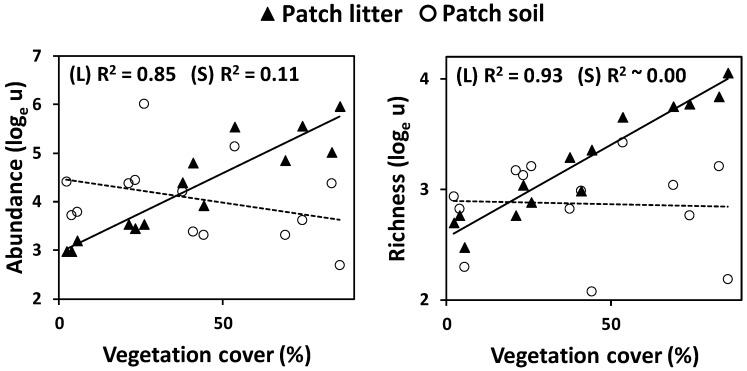

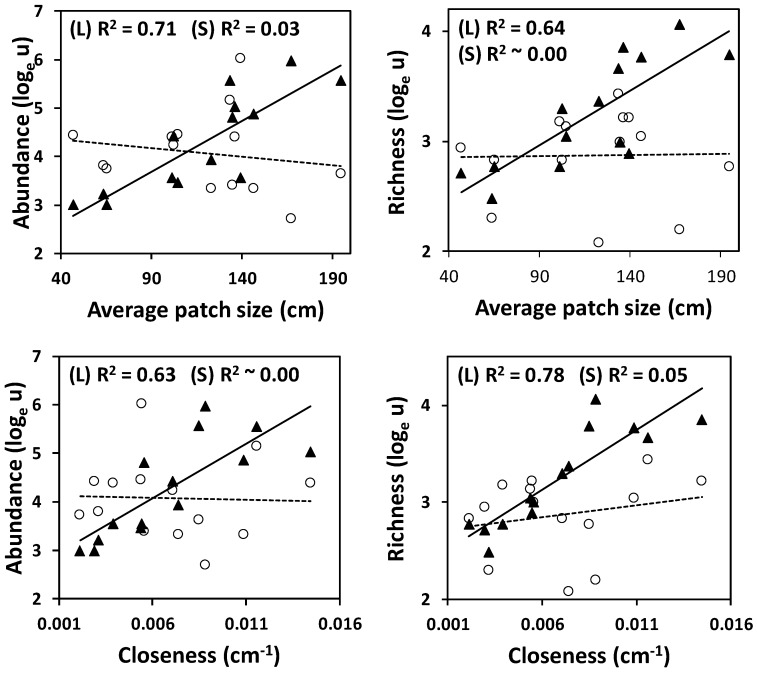
Relationships between log-scaled ground arthropod abundance (left panels) and richness (right panels) and landscape vegetation pattern metrics (vegetation cover, average patch size, and patch closeness) as a function of the soil layer: Litter (L) and soil (S). Continuous lines represent statistically significant linear regressions (*p* ≤ 0.05) and dashed lines represent not significant regressions between the respective log-transformed response variable and the explanatory variable for each soil layer.

At the landscape (plot) scale, the abundance and species richness of ground arthropods, both in patches as in interpatches, were positively and exponentially related to vegetation cover, patch size and closeness (Figure 1). Both abundance and species richness were significantly higher in patches than in interpatches (Figure 1; Table 1). The positive effect of vegetation cover and closeness on soil fauna depended on the microsite type, with a higher increase in arthropod abundance and richness in interpatches than in patches as a consequence of the increase in closeness (Table 1), and a higher increase in abundance in interpatches than in patches as a consequence of the increase in vegetation cover. For the patch microsites, arthropod species richness was higher in the litter than in the soil layer, yet arthropod abundance was similar in both microhabitats (Figure 2; Table 1). The influence of vegetation pattern was observed for the arthropod community of the litter layer, which showed exponentially increasing abundance with increasing *Vc*, *Ps* and *Cl* values, but not for the community of the soil layer (Figure 2), as captured by the significant interactions between the soil layer factor and either *Vc*, *Ps* or *Cl* (Table 1).

According to the partial correlation analyses, vegetation patch closeness was the most important landscape driver of ground arthropod abundance in the interpatch microhabitat, being very significantly correlated with interpatch arthropod abundance also when the individual or joint effect of *P_S_* and *V_C_* were removed (Table 2). Closeness was also the most important individual driver of interpatch species richness, yet its effect slightly vanished once both *V_C_* and *P_S_* effects were removed. Vegetation cover was also significantly correlated with interpatch arthropod abundance and species richness after controlling for *P_S_*, but not after controlling for *Cl* (Table 2).

For the patch microhabitat, the individual influence of each vegetation pattern variable on the abundance and richness of ground fauna was more diffuse than for the interpatches. Patch size and arthropod abundance correlation was still significant once the influence of *Cl* was removed, and marginally significant after controlling for either *Vc* or both *Vc* and *Cl*. Conversely, the correlations between arthropod abundance and either *Vc* or *Cl* became non-significant once the individual or joint effect of the other descriptors was removed (Table 2). The positive effect of *V_C_* on the arthropod species richness in patches was still significant once the effect of either *P_S_* or *Cl* were removed, while the effects of *Cl* and *Ps* disappeared after controlling for *Vc*. The positive effect of *Cl* on species richness was still significant when the influence of *P_S_* was removed, while the positive effect of *Ps* was only marginally significant after controlling for *Cl* (Table 2).

**Figure 3:**
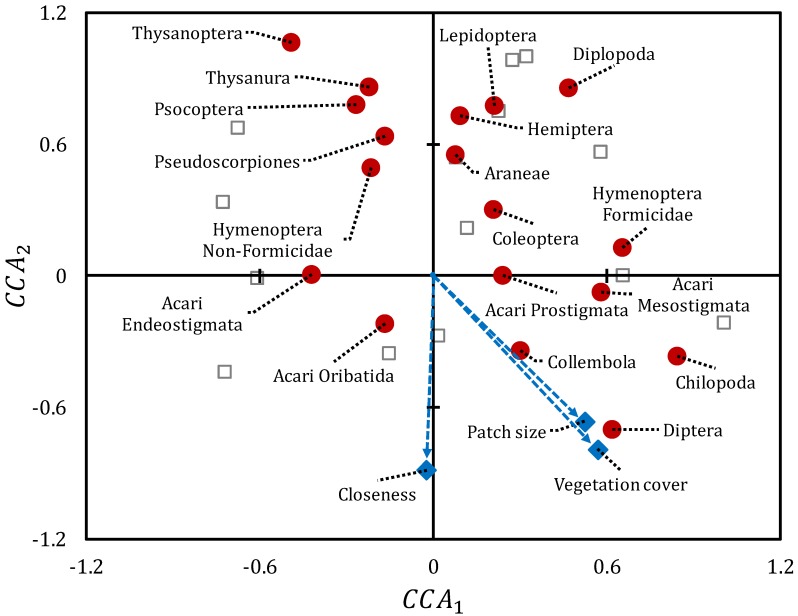
Variation in the composition of ground arthropod communities sampled in plots with different vegetation spatial pattern, according to the ordination resulting from Canonical Correspondence Analysis (CCA). Open gray squares: plots; filled red circles: taxa; filled blue diamonds: vegetation pattern variables; blue arrows: direction of increasing values; Vc: vegetation cover; Ps: average size of patches; Cl: closeness between patches. Acari Endeostigmata (R^2^ = 0.58); Acari Oribatia (R^2^ = 0.46); Acari Prostigmata (R^2^ = 0.47); Acari Mesostigmata (R^2^ = 0.66); Araneae (R^2^ = 0.35); Chilopoda (R^2^ = 0.56); Coleoptera (R^2^ = 0.13); Collembola (R^2^ = 0.69); Diptera (R^2^ = 0.59); Diplopoda (R^2^ = 0.45); Hemiptera (R^2^ = 0.47); Hymenoptera Formicidae (R^2^ = 0.29); Hymenoptera non-Formicidae (R^2^ = 0.16); Lepidoptera (R^2^ = 0.14); Psocoptera (R^2^ = 0.48); Pseudoscorpiones (R^2^ = 0.20); Thysanoptera (R^2^ = 0.33); Thysanura (R^2^ = 0.29).

The CCA revealed the general influence of the vegetation spatial pattern on the plot composition of high-level taxa. The best fit model was achieved by considering *Vc* (pseudo-*F* = 3.37; *p* = 0.003), *Ps* (pseudo-*F* = 2.76; *p* = 0.013), and *Cl* (pseudo-*F* = 1.98; *p* = 0.069; Figure 3), with *Vc* and *Ps* being the most relevant variables. The constraining process explained 44.8% of total data dispersion, and the dimensions CCA_1_ and CCA_2_ explained, respectively, the 55% and the 38% of the constrained variation in arthropod composition. Some arthropod taxa considered as “specialized to soil niches”, such as mites (Acari Endeostigmata and Mesostigmata), centipedes (Chilopoda), springtails (Collembola) and fly larvae (Diptera), showed higher abundance where patch size and vegetation cover were also higher. Other important groups for soil niches, such as Acari Oribatida and Prostigmata, related to plots with higher patch closeness. On the contrary, Diplopoda related to plots with low closeness. Some epigeic taxa, such as true bugs (Hemiptera), spiders (Araneae), barkflies (Psocoptera), and thrips (Thysanoptera) weakly related to plots with low vegetation cover, patch size, and closeness (Figure 3). Hymenoptera (mainly Formicidae), moth larvae (Lepidoptera) and silverfish (Thysanura) showed very poor correlation with vegetation pattern variables. The final CCA disregarded data of Opilionida, Protura, Diplura, and Embiidina due to rare occurrence.

### 3.2. Sample-Based Analyses

At the individual-patch scale, arthropod abundance and richness were mostly explained by patch size (*P_S(i)_*), which was significantly correlated to both abundance and richness once the explanatory effect of distance (*D_P-N_*) and/or size (*N_S_*) of the neighboring patches were removed. Conversely, *D_P-N_* and *N_S_* did not show any significant correlation after controlling for the effect of *P_S(i)_*, yet *D_P-N_* showed significant correlations with arthropod abundance and richness when only the effect of *N_S_* was controlled (Table 3).

**Figure 4:**
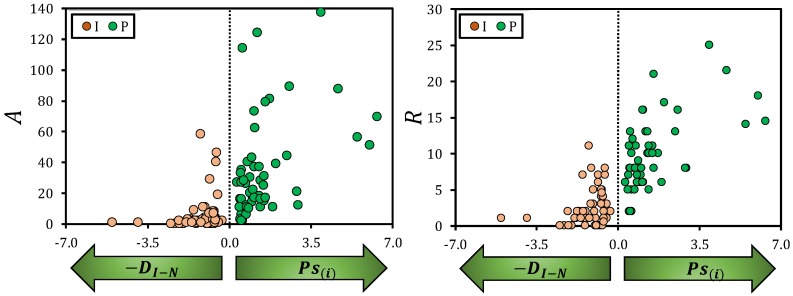
Spatial distribution of the abundance (*A*, left panel) and species richness (*R*, right panel) of ground arthropods observed in interpatches (*I*) and patches (*P*) sampling points in a semiarid steppe as a function of the average distance between the interpatch points and the neighbor patches (represented as negative values: −*D_I-N_*) and the patch size (*P_S(i)_*), both metric measured in meters. The green arrows indicate the direction of increase for which each pattern metric.

The spatial distribution of the abundance and species richness of ground arthropods largely responded to the patch-interpatch structure of the target semiarid steppe landscape (Figure 4). On the one hand, both arthropod abundance (*A*) and species richness (*S*) found in interpatch points increased with decreasing distance to vegetation patches. For small distances, interpatch *A* and *R* were similar to the *A* and *R* values found for small vegetation patches (Figure 4, left panel). On the other hand, *A* and *R* of ground arthropods living under vegetation patches fast increased with increasing patch size (Figure 4, right panel). The combined response of patch and interpatch communities defined a sigmoid curve (Figure 4), indicating maximum *A* and *R* under well-developed vegetation patches that rapidly decreases around the patch-interpatch transition and further decreases towards a minimum with increasing distance to vegetation patches (Supplementary materials, Figure S4).

## 4. Discussion

### 4.1. Ground Arthropods as a Function of Vegetation Pattern

Our findings show that the vegetation pattern of dryland ecosystems control the abundance, richness and spatial distribution of ground meso-and microarthropods. Strong relationships between ground organisms and vegetation amount, diversity, and heterogeneity have been demonstrated for many types of ecosystems worldwide [27,57,58]. Here we provide evidence that dryland communities of ground meso and microarthropods strongly respond to changes in the spatial pattern of vegetation. More specifically, our findings show that arthropod communities living under vegetation patches mostly respond to patch size, and the communities from bare-soil interpatches mostly respond to the average distance between patches, regardless of the potential concurrent changes in vegetation cover.

The harsh environmental conditions prevailing in semiarid ecosystems (e.g., extreme dryness, radiation, and temperature), the sensitivity of ground arthropods to the environmental filter resulting from such conditions, and the protection and resources provided by plants [22] could jointly explain the control exerted by the vegetation pattern over the dryland communities of ground arthropods. For example, mite quantities in dryland soils have been found to strongly depend on the amount of soil organic matter and on the soil structure [38], properties that are largely influenced by the spatial pattern of the vegetation [4,5,59]. Our results indicate that total vegetation cover is a critical factor modulating the ground meso- and microarthropods communities in drylands, which is in agreement with previous findings reported for different groups of ground arthropods [9,34,60,61]. We could, therefore, expect that decreasing vegetation cover may lead to strong direct impacts on ground communities, a response already reported for spiders and microorganisms [61,62]. However, the facilitative influence of vegetation cover on the development of ground fauna cannot solely explain abundance and diversity of ground meso-and microarthropods in dryland systems. We found many more individuals and species of ground arthropods in vegetation patches than in interpatches, yet we found no species that occurred only in the interpatch microhabitat. Furthermore, relatively small distances between the vegetation patches appeared to be sufficient to isolate the communities living under those patches, with interpatch abundance dropping to few individuals of very few species at points located 2–3 m apart from the neighboring vegetation patches. In fact, independently of the effect of plant cover, the proximity between patches increased abundance and species richness of interpatch ground arthropods, and patch size marginally increased arthropod abundance under vegetation patches.

Several recent studies emphasized that local factors (e.g., patch size and patch shape) could be more important than landscape-scale factors such as patch isolation [61,63]. Our results highlight that the spatial arrangement of vegetation is also important to explain the spatial distribution of ground arthropods. In drylands, the local protection and resources provided by the plant patch are critical to maintaining viable populations, while the level of isolation modulated by the distance between patches imposes restrictive conditions on individual survival and controls the spatio-temporal dynamics of meso-and microarthropod communities. Interestingly, the taxonomic composition of ground arthropods was greatly affected by vegetation, but it was poorly affected by patch closeness. These results suggest that the isolation imposed by the distance between patches tended to impact all groups of soil meso- and microarthropods in a similar way, while patch size and vegetation cover drove the taxonomic composition. However, the fact that larger patches favored some taxa over other groups indicates that the influence of vegetation spatial pattern on ground meso-and microarthropods goes beyond the quantitative variation in abundance and species richness, affecting also the taxonomic structure of the ground arthropod communities.

The transitional zone between patch and interpatch (bare-soil) areas exhibited an important change in ground arthropod abundance and species richness, suggesting that edge effect [64,65] could also be a key process in community organization at the microhabitat scale. Furthermore, the fact that ground meso-and micro-arthropod are constrained to zones located under or very close to vegetation patches (relative isolation), suggests that patch size and the distance between patches might modulate the diversity of ground arthropod species in agreement with the theory of island biogeography [66]. This hypothesis has been previously used to explain the regional species composition of ground arthropods in tropical forests [19], which concerns long distances, and could also frame the ground meso-and microarthropod response to patchy landscapes. Arthropod communities, mainly micro-arthropods, often exhibit a short-range dependence on vegetation and local conditions [21,67,68,69], being subjected to relative isolation at very short distances. Our results suggest that large patches act as “core” islands in the landscape, maintaining the diversity of ground arthropods in these ecosystems and providing propagules for the colonization of new patches. This finding points to the potential of large vegetation patches for recovering degraded drylands by improving the conservation and colonization capacity of the ground arthropod communities. The result also stresses the importance of both local and landscape conditions to maintain diverse soil communities [63].

We found that the strong response of the patch ground arthropods to the variation in vegetation cover and pattern mostly relied on the arthropod communities living in the litter layer. In addition to the recognized decreasing pattern in the vertical distribution of resources (e.g., organic material) and niches throughout the litter-soil column [25,70,71], microhabitat conditions are also particularly important to explain the vertical response of ground arthropods. Protection increases and stressful environmental conditions attenuates down through the vertical ground layers [27], buffering the impact of vegetation change on the under-patch soil communities, as compared with the litter communities. The stratification of faunal response to vegetation pattern is not common to all ecosystems and seasons and seems to be dependent on the occurrence of general or seasonal harsh conditions [9,19,72].

It is well-known that changes in dryland vegetation cover and pattern are tightly linked to changes in ecosystem functions [2,7,73]. Given the crucial role played by ground arthropods in multiple soil processes [23,60,74,75], the dependence of dryland ground arthropods on the vegetation spatial pattern here presented contributes to explain the strong pattern-function relationships exhibited by dryland ecosystems. Accordingly, it could be expected that environmental pressures leading to decreasing vegetation cover and/or connectivity of vegetation patches result in additional indirect impact through the loss of key ground-arthropod taxa, amplifying the overall adverse effect on ecosystem functioning. We found that the taxa that were most positively affected by the amount of vegetation are generally considered as specialized in edaphic niches. This result suggests that increasing abundance of these taxa depends on the development of habitats with certain minimum availability of resources [76], which allows for association of their abundance and key ecological processes, and qualifies them as ecological indicators [30,77,78]. Our study did not test such a hypothesis, but our findings point to the potential of some niche specialized groups (for instance larvae of Diptera, Chilopoda, and Collembola) for bioindication of soil health in Mediterranean drylands.

### 4.2. Diversity of Meso-and Microarthropods in Mediterranean Drylands

We found a relatively high abundance and diversity of ground meso- and microarthropods in the studied Mediterranean dryland steppe, with high dominance of Acari groups. These findings agree with previous results from other dryland ecosystems, such as those reported for Australian semi-arid ecosystems [38], for a Chihuahuan desert watershed [79], and for the biological crust in Mexican desert scrub [80], all works describing a great dominance of Acari (mainly Prostigmata) in the soil micro-arthropod communities. Despite the abiotic limitations that characterize dryland ecosystems, the soil fauna in these ecosystems is diverse and includes key groups, such as prostigmatid mites, that play a critical role in regulating decomposition and mineralization processes by feeding on fungi and nematodes, and that may remain active under very dry conditions [23]. The dominance of mites could have been overexpressed in our study due to the relatively dry and warm conditions of the sampling period (late spring-early summer), yet these conditions extend over large periods in the Mediterranean drylands and are expected to increase their importance in the future as a consequence of the on-going climate change [81]. In contrast with our results, a comprehensive review of the composition of the terrestrial arthropod communities in arid systems of SE Spain [21] reported that litter and belowground arthropod communities were dominated by ants and Coleoptera. In our study, we used modified Berlese-Tülgren funnels, a method intended to sample small arthropods, while the review by Reference [21] reported on data obtained mostly from pitfall traps, which are particularly efficient in sampling active ground-dwelling arthropods, yet they may under-represent small arthropods with low mobility that live in the soil. Our data thus provide complementary information on the litter and belowground assemblages of soil arthropods, contributing to increasing the general understanding of the biological diversity and trophic interactions of the soil fauna in Mediterranean drylands.

## 5. Conclusions

Vegetation cover, patch size, and between-patch distance exert a strong effect on the abundance and species richness of ground meso-and microarthropods in dryland steppes. On average, patch microhabitats sustain six times larger and four times richer communities of ground arthropods than interpatch microhabitats. Patch and interpatch communities also differ in their response to plant pattern, with patch communities being mostly controlled by patch size and interpatch communities mostly controlled by the average distance between patches. The combined influence of both microhabitats results in maximum abundance and diversity at the core of the vegetation patches that rapidly decrease around the patch-interpatch transition, and further decrease towards a minimum with increasing distance to the patches. The dependence of the ground communities on the vegetation pattern can be explained as a consequence of habitat and resource availability, reflected in the role played by vegetation cover and patch size, and the potential isolation of favorable microhabitats resulting from the spatial arrangement of vegetation patches and bare-soil areas. Our results suggest that scenarios in which vegetation patches are drastically reduced in size and proximity can lead to the loss of the habitat conditions necessary for sustaining diverse ground communities in drylands, with unknown consequences for soil functions; a topic that demands further research.

## Figures and Tables

**Table 1:** Results (*F* and *p* values) of ANCOVAs on landscape-scale (plot) abundance and species richness of ground arthropods as a function of the factor Microsite, *M* (patch and inter-patch), or Soil layer, *L* (litter and soil) and of different vegetation pattern covariables: *V_C_*, Vegetation cover; *P_S_*, average patch size; *Cl*: closeness (inverse of distance between patches); *n* = 14; *, **, ***: significant at 5%, 1%, and 0.1%, respectively.

ANCOVAs Microsite (*M*)	Abundance (log)		Richness (log)		ANCOVAs Soil Layer (*L*)	Abundance (log)		Richness (log)	
	*F*	*p*	*F*	*p*		*F*	*p*	*F*	*p*
*V_C_*	28.5	<0.001 ***	30.5	<0.001 ***	*V_C_*	6.7	0.016 **	14.2	<0.001 ***
*M*	48.1	<0.001 ***	112.7	<0.001 ***	*L*	0.7	0.409	9.8	0.005 **
*V_C_:M* ^‡^	5.0	0.035 *	--	--	*V_C_:L*	22.9	<0.001 ***	16.3	<0.001 ***
	*F*	*p*	*F*	*p*		*F*	*p*	*F*	*p*
*P_S_*	16.2	<0.001 ***	14.3	<0.001 ***	*P_S_*	6.22	0.019 *	7.9	0.010 **
*M*	34.4	<0.001 ***	79.8	<0.001 ***	*L*	0.6	0.461	7.0	0.014 *
*P_S_*:*M*^‡^	--	--	--	--	*P_S_:L*	12.4	0.002 **	7.3	0.012 *
	*F*	*p*	*F*	*p*		*F*	*p*	*F*	*p*
*Cl*	52.4	<0.001 ***	47.8	<0.001 ***	*Cl*	6.7	0.016 *	15.7	<0.001 ***
*M*	74.9	<0.001 ***	156.1	<0.001 ***	*L*	0.5	0.483	8.4	0.008 **
*Cl:M*	13.2	0.001 **	5.0	0.035 *	*Cl:L*	7.7	0.010 *	7.0	0.014 *

Notes: ^‡^ In case of non-significant effect of the interaction between the factor and the covariable (--), *F* and *p* values for the main factor and covariable correspond to an ANCOVA model without interaction term.

**Table 2:** Partial correlation coefficients (*p*) and *p*-values for the relationships between landscape-scale vegetation pattern variables and species richness and abundance of ground arthropods found in vegetation patches and inter-patches. *V_C_*: Vegetation cover; *P_S_*: Average patch size; *Cl*: Average closeness between patches (inverse of respective average distance); ○: marginally significant; *, **, ***: significant at 5%, 1%, and 0.1%, respectively.

Faunal Variable	Patches				Inter-Patches		
	Vegetation Variable	Controlled By	*p*	*p*-Value	Controlled By	*p*	*p*-Value
Abundance	*Vc*	*Ps*	0.007	0.980	*Ps*	0.644	0.017 *
		*Cl*	0.313	0.297	*Cl*	−0.065	0.831
		*Ps; Cl*	−0.301	0.341	*Ps; Cl*	−0.154	0.633
	*Ps*	*Vc*	0.482	0.095*○*	*Vc*	−0.219	0.471
		*Cl*	0.578	0.038 *	*Cl*	0.034	0.913
		*Vc; Cl*	0.574	0.051*○*	*Vc; Cl*	0.143	0.657
	*Cl*	*Vc*	0.085	0.782	*Vc*	0.728	0.005 **
		*Ps*	0.214	0.482	*Ps*	0.843	<0.001 ***
		*Vc; Ps*	0.364	0.245	*Vc; Ps*	0.719	0.008 **
Richness	*Vc*	*Ps*	0.734	0.004 **	*Ps*	0.564	0.045 *
		*Cl*	0.616	0.025 *	*Cl*	−0.020	0.949
		*Ps; Cl*	0.385	0.216	*Ps; Cl*	−0.079	0.806
	*Ps*	*Vc*	−0.052	0.865	*Vc*	−0.179	0.558
		*Cl*	0.525	0.065*○*	*Cl*	0.035	0.910
		*Vc; Cl*	0.069	0.830	*Vc; Cl*	0.084	0.794
	*Cl*	*Vc*	0.266	0.379	*Vc*	0.571	0.041 *
		*Ps*	0.709	0.007 **	*Ps*	0.725	0.005 **
		*Vs; Ps*	0.270	0.396	*Vc; Ps*	0.556	0.060○

**Table 3:** Partial correlation coefficients (*p*) and *p*-values for the relationships between spatial pattern descriptors and species richness and abundances of ground arthropods found in vegetation patches. Pattern descriptors; *P_S(i)_*: size of the patch; *N_S_*: average size of neighbor patches; *D_P-N_*: Average distance between the target patch and its neighbor patches; ○: marginally significant; *, **, ***: significant at 5%, 1% and 0.1%, respectively.

Abundance				Richness			
Veg. Variable	Controlled By	*p*	*p*-Value	Veg. Variable	Controlled By	*p*	*p*-Value
*P_S(i)_*	*N_S_*	0.48	<0.001 ***	*P_S(i)_*	*N_S_*	0.47	<0.001 ***
	*D_P-N_*	0.34	0.011 *		*D_P-N_*	0.46	<0.001 ***
	*N_S_*; *D_P-N_*	0.58	<0.003 **		*N_S_*; *D_P-N_*	0.60	0.001 **
*N_S_*	*P_S(i)_*	−0.19	*0.155*	*N_S_*	*P_S(i)_*	0.01	0.919
	*D_P-N_*	−0.08	0.569		*D_P-N_*	0.17	0.207
	*P_S(i)_*; *D_P-N_*	0.05	0.721		*P_S(i)_*; *D_P-N_*	0.252	0.063○
*D_P-N_*	*P_S(i)_*	0.18	0.190	*D_P-N_*	*P_S(i)_*	−0.18	0.189
	*N_S_*	0.37	0.005 **		*N_S_*	−0.28	0.041 *
	*P_S(i)_*; *N_S_*	0.20	0.134		*P_S(i)_*; *N_S_*	−0.14	0.306

## Supplementary Materials

The following are available online at https://www.mdpi.com/2075-4450/11/1/59/s1, Figure S1: Semi-arid Mediterranean steppes in the study area, Cabezo de la Plata, Murcia, Spain; Figure S2: Schematic illustration of a sampling plot; Figure S3: Relative abundance of ground arthropods taxa found in semi-arid Mediterranean steppe areas in Southeast Spain; Figure S4: Abundance and species richness of ground arthropods found in interpatches and patches as a function of the average distance to the nearest neighbor patches; Table S1: List of taxa and number of morpho-species found in the litter and top-soil layers of fourteen plots of Mediterranean drylands in Southeast Spain.
