# Supplementary material for: Vegetation Pattern Modulates Ground Arthropod Diversity in Semi-Arid Mediterranean Steppes
Source: Insects. 2020 Jan 18;11(1):59. doi: 10.3390/insects11010059 (PMC7023303; doi:10.3390/insects11010059)
Supplement: Supplementary file 1 [file insects-11-00059-s001.pdf]

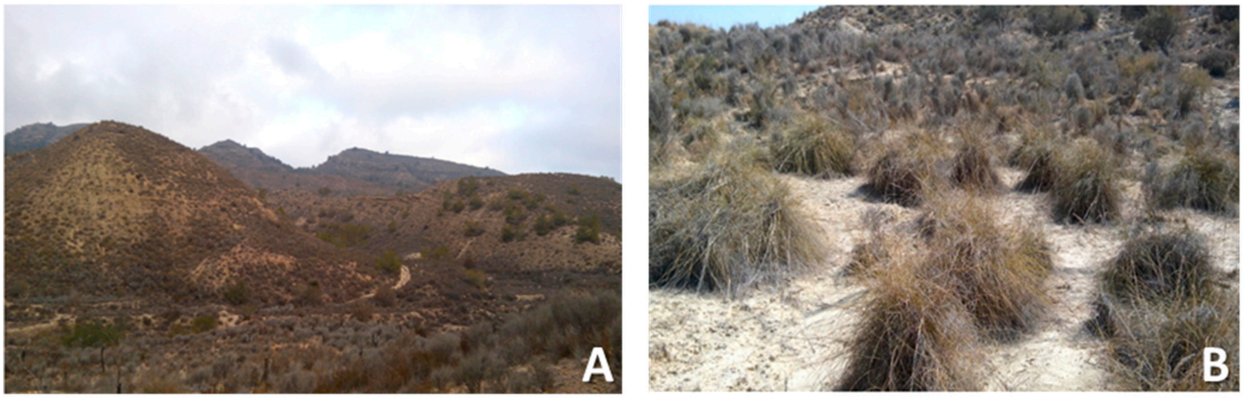

Figure S1. (**A**) Semi-arid Mediterranean steppes in the study area, Cabezo de la Plata, Murcia, Spain. (**B**) Detailed view of the steppe landscape in the study area, with a mosaic of vegetation patches dominated by the tussock grass *Stipa tenacissima*, L., and bare-soil inter-patches.

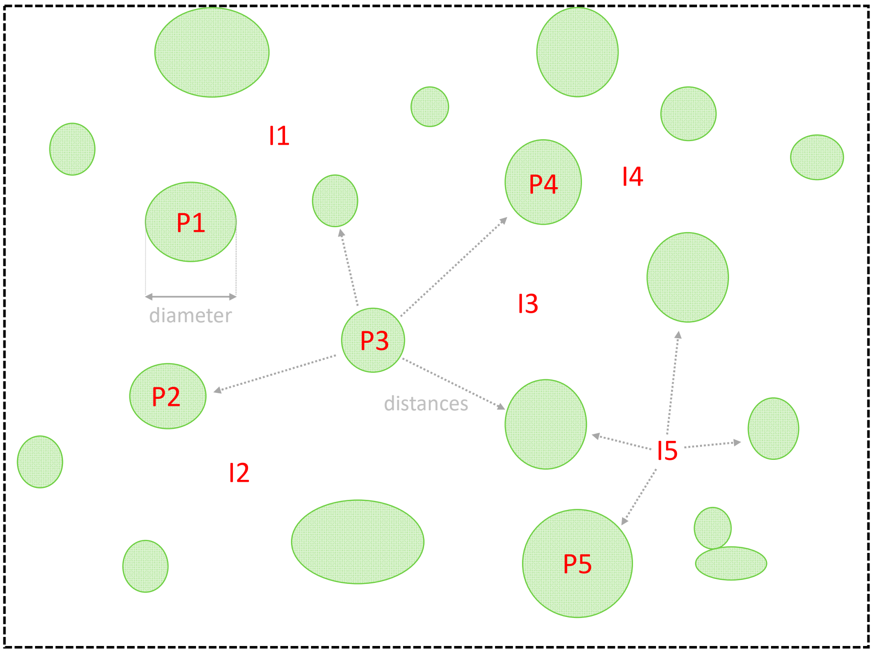

Figure S2. Schematic illustration of a sampling plot (~400 m^2), with vegetation patches (green patches) on a bare-soil matrix. Each letter followed by a number (red labels) represents a soil-fauna sampling point, with sampling patches represented as P1…P5, and sampling interpatches represented as I1…I5. For each sampling point, the distance to each of the four nearest neighbor patches was measured. Patch size was measured as patch diameter (cm).

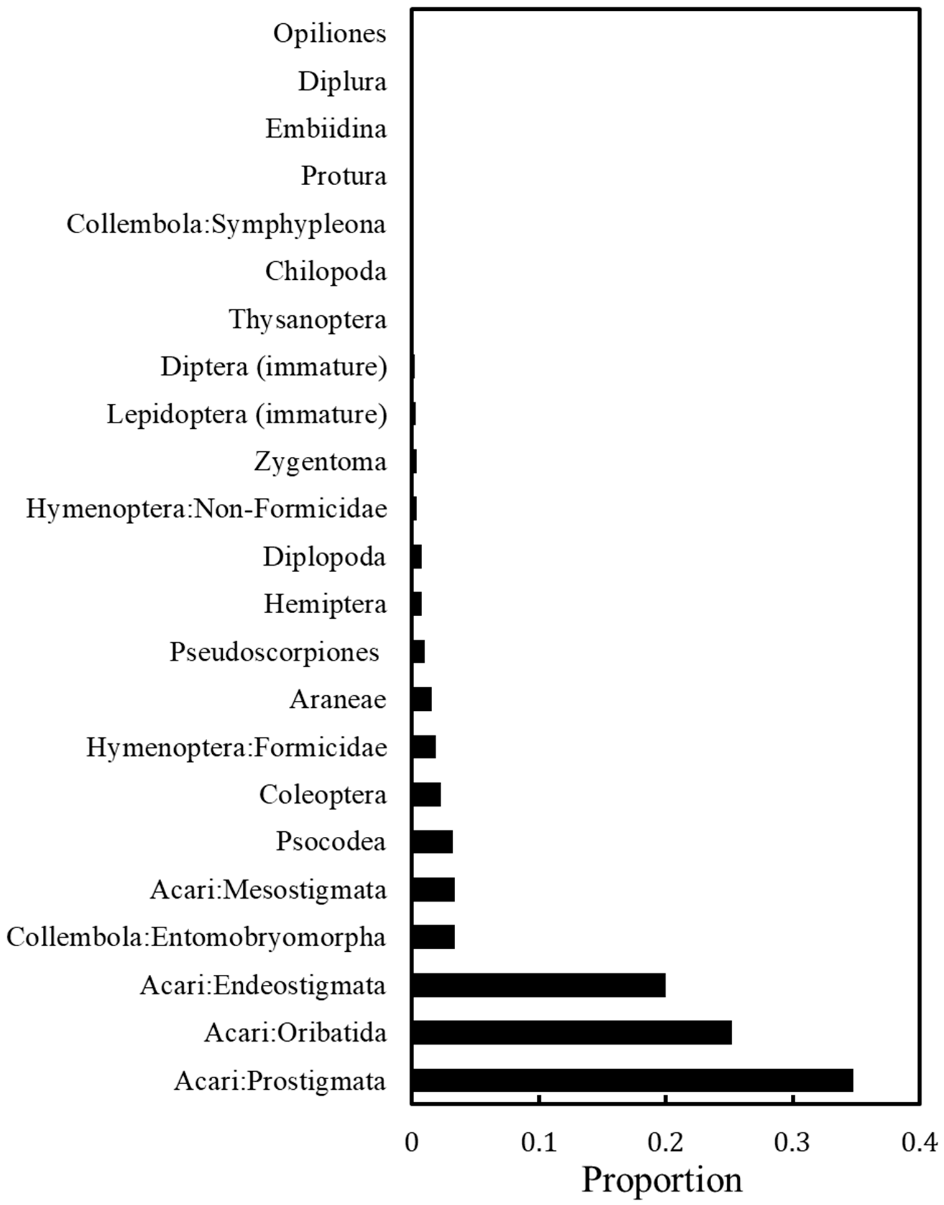

Figure S3. Relative abundance of ground arthropods taxa found in semi-arid Mediterranean steppe areas in Southeast Spain.

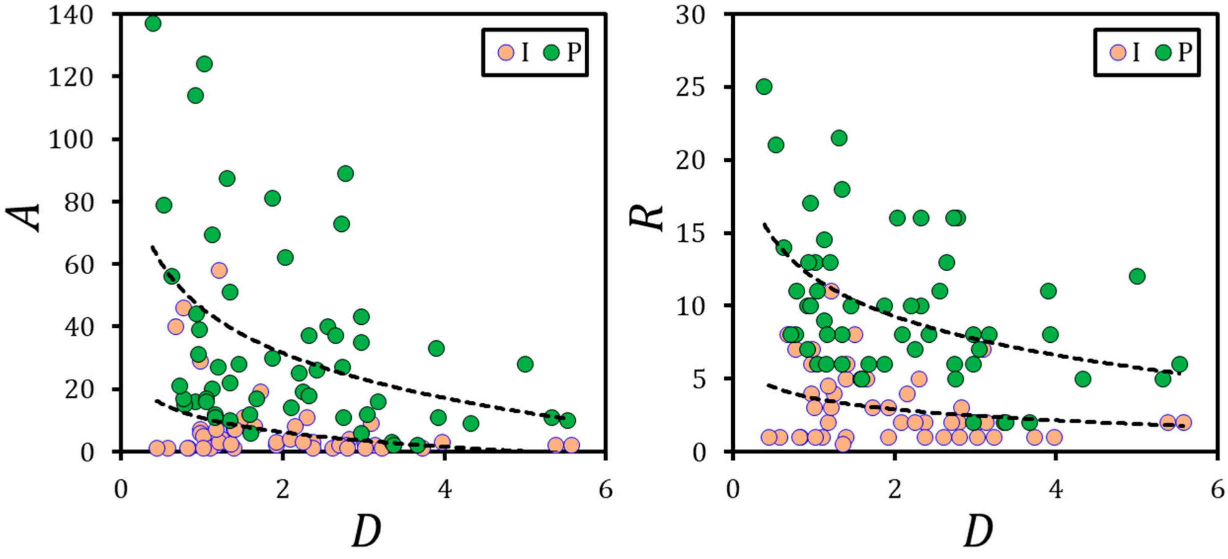

Figure S4. Abundance (A) and species richness (R) of ground arthropods found in interpatches (I) and patches (P) as a function of the average distance (D) to the four nearest neighbor patches in semi-arid Mediterranean steppe areas in Southeast Spain. Dashed lines are exponential fits.

Table S1. List of taxa and number of morpho-species found in the litter and top-soil layers of fourteen plots of Mediterranean steppe areas in Southeast Spain. Indet Superf—Indeterminate Superfamily; Indet Fam: Indeterminate Family; Indet Genera: Indeterminate Genera; All Lepidoptera and most of Diptera are immatures.

List of taxa							Number of morphospecies
**Class Arachnida**							
	**Ord. Acari**						
		**Clade Endeostigmata**					
			Pachygnathina				
				Alycoidea			
					Nanorchestidae	Speleorchestes	1
		**Clade Mesostigmata**					
			Dermanyssina				
				Ascoidea			
					Ascidae		1
				Dermanyssoidea			
					Laelapidae		1
					Macronyssidae		1
					Indet Fam		2
				Eviphidoidea			
					Indet Fam		1
				Veigaioidea			
					Veigaiidae		1
			Ixodida				
				Ixodoidea			
					Ixodidae		1
			Parasitina				
				Parasitoidea			
					Parasitidae		4
			Uropodina				
				Uropodoidea			
					Uropodidae		1

**Clade Oribatida**					
	Astigmatina				
		Acaroidea			
			Acaridae	*Histiogaster*	1
				Tyrophagus	1
				Indet Genera	4
		Histiostomatoidea			
			Histiostomatidae		1
			Indet Fam		1
	Brachypylina				
		Ameroidea			
			Ctenobelbidae	*Ctenobelba*	1
		Carabodoidea			
			Carabodidae	*Carabodes*	1
		Damaeoidea			
			Belbodamaeidae	Porobelba	1
		Eremaeoidea			
			Eremaeidae	*Erememaeus*	1
				Indet Genera	1
		Gustavioidea			
			Astegistidae		1
			Xenillidae	*Xenillus ninfa*	1
		Licneremaeoidea			
			Scutoverticidae	Scutovertex sculptus	1
				Indet Genera	1
		Plateremaeoidea			
			Gymnodamaeidae	*Gymnodameus*	2
	Desmonomata				
		Crotonioidea			

		Nothridae	*Nothrus*	1
Enarthronota				
	Brachychthonioidea			
		Brachychthoniidae	*Brachychthnius*	1
			Indet Genera	2
Palaeosomata				
	Ctenacaroidea			
		Aphelacaridae	*Aphelacarus*	1
Palaeosomata	Protoplophoroidea	Cosmochthoniidae		1
		Indet Fam		1
Poronata				
	Cerarozeroidea			
		Humerobatidae		1
	Ceratozetoidea			
		Chamobatidae		1
	Oripodoidea			
		Oribatulidae	*Zygoribatula*	1
			Oribatula	1
		Scheloribatidae	*Scheloribates*	1
			Indet Genera	2
	Phenopelopoidea			
		Phebopelopidae	*Eupelops*	2
			Indet Fam	1
**Clade Prostigmata**				
Anystina				
	Anystoidea			
		Anystidae	*Anystis*	2
			Indet Genera	4
		Indet Fam		2
	Caeculoidea			
		Caeculidae	*Andocaeculus*	2

			Caeculus	2
			Indet Fam	1
	Erythraeoidea			
		Erythraeidae		1
		Indet Fam		1
	Trombidioidea			
		Microtrombidiidae		1
		Trombidiidae		1
		Chyletidae		1
		Indet Fam		1
Eleutherengona				
	Tetranychoidea	Indet Fam		1
Eupodina				
	Bdelloidea			
		Bdellidae	*Bdella*	1
			Indet Genera	1
	Bdelloidea	Cunaxidae	*Cunaxa*	1
			Indet Genera	3
	Eupodoidea			
		Eupodidae		3
		Indet Fam		3
		Rhagidiidae	*Rhagidia*	1
			Indet Genera	3
		Indet Fam		3
	Tydeoidea			
		Tydeidae	*Bimichaelia*	1
			Indet Genera	3
		Indet Fam		1
Heterostigmatina				
	Tarsocheyloidea			
		Tarsocheylidae		1

	Parasitengona				
		Erythraeoidea			
			Erythraeidae		1
			Indet Fam		1
	Raphignathina				
		Cheyletoidea			
			Cheyletidae		1
			Indet Fam		1
		Raphignathoidea			
			Caligonellidae		3
			Caligonellidae	*Caligonella*<br>*Molothrognathus*	1
			Raphignathidae	*Rhaphignatus*	3
			Stigmaeidae		1
			Indet Fam		4
**Ord. Araneae**					
			Ctenizidae		1
			Dictynidae		1
			Dictynidae		1
			Gnaphosidae		4
			Immature		2
			Leptonetidae		1
			Linyphiidae		1
			Linyphiidae		1
			Lycosa immature		2
			Mysmenidae		2
			Oonopidae		2
			Palpimanidae		1
			Philodromidae		1
			Prodidomidae	*Zimirina*	1
			Salticidae		2
			Scytodidae	*Scytodes thoracica*	1

				Sicariidae		1
				Theridiosomatidae	*Theridiosoma*	1
	**Ord. Opiliones**					
		Clade Palpatores				
			Phalangioidea			1
	**Ord. Pseudoscorpiones**					
				Atemnidae	*Diplotemnus insolitus*	1
				Cheiridiidae	*Apocheiridium ferum*	1
				Cheliferidae	*Rhacochelifer*	1
				Indet. Fam.		1
				Geogarypidae	*Geogarypus nigrimanus*	1
				Olpiidae	*Minniza iberica*	1
**Class Entomognata**						
	**Ord. Diplura**					
				**Japygidae**		**1**
	**Ord. Collembola**					
		**Clade Entomobryomorpha**				
			Entomobryidae	Entomobryidae	*Entomobrya*	2
			Entomobryidae	Entomobryidae	*Entomobrya multifasciata*	1
			Entomobryidae	Entomobryidae	*Seira*	1
			Entomobryidae	Entomobryidae	*Sinella*	1
			Entomobryidae	Entomobryidae	*Willosia*	3
			Entomobryidae	Entomobryidae	Indet Gen	3
			Entomobryoidea	Isotomidae	Indet Gen	1
		**Clade Symphypleona**				
				Dicyrtomidae	*Dicyrtomina ornata*	2
**Class Insecta**						

**Ord. Coleoptera**				
		Carabidae		4
		Chrysomelidae		3
		Curculionidae		2
		Dermestidae		1
		Elateridae		1
		Endomychidae		1
		Ptinidae		3
		Scarabeidae		2
		Staphylinidae		5
		Tenebrionidae		2
**Ord. Diptera**				
		Cecidomyiidae		2
		Chironomidae		2
		Ptychopteridae		1
		Scatopsidae		1
		Sciaridae		1
		ND		6
**Ord. Embiidina**				
		Oligotomidae	*Haploembia*	1
**Ord. Hemiptera**				
**Clade Auchenorrhyncha**				
	Cicadoidea			
		Cicadellidae		3
		Cicadidae		1
**Clade Heteroptera**				
	Cimicoidea			
		Anthocoridae		1
	Pentatomoidea			
		Cydnidae		3
	Lygaeoidea			

				Lygaeidae		2
		**Clade Sternorrhyncha**				
			Aleyrodoidea			
				Aleyrodidae		1
			Coccoidea			
				Indet Fam		5
			Indet Superfam			1
	**Ord. Hymenoptera**					
				Formicidae	*Brachymyrmex*	1
					Camponotus	1
					Camponotus (Myrmosericus) *cruentatus*	1
					Crematogaster (Acrocelia)	1
					Pheidole	2
					Solenopsis	3
				Não-Formicidae		7
	**Ord. Lepidoptera**					
				Indet Fam		6
	**Ord. Protura**					
			Eosentomoidea	Eosentomidae		2
	**Ord. Psocodea**					
				Liposcelididae	Liposcelis	1
				Trogiidae	Lepinotus	1
	**Ord. Thysanoptera**					
				Adiheterothripidae		1
				Phlaeothripidae		2
				Thripidadae		1
	**Ord. Zygentoma**					
				Lepismatidae	Ctenolepisma	3
**Class Chilopoda**						

**Ord. Geophilomorpha**		
	Indet Fam	2
**Ord. Scutigeromorphae**		
	Indet Fam	1
**Class Diplopoda**		
**Ord. Chordeumatida**		
	Indet Fam	1
**Ord. Julida**		
	Indet Fam	1
**Ord. Polyxenida**		
	Indet Fam	3

Reference (List of sources used to support taxonomic identification)

1. Arnett, R.H., 2000. American Insects: Handbook of the Insects of America North of Mexico. CRC Press, Boca Raton, Florida
2. Barrientos, J.A., 2006. III Curso practico de aracnologia: jornadas sobre taxonomia de arácnidos ibéricos. Laboratorio de Entomología, Facultad de Cienicas, Universidad de Córdoba y Grupo Ibérico de Aracnología, Sociedad Entomológica Aragonesa, Córdoba, España.
3. Balogh, J., 1961. Identification keys of world oribatid (Acari) families and genera. Acta Zoologica Academiae Scientiarum Hungaricae 7:243-344
4. Bei-Bienko, G.Y.A., Blagoveshchenskii, D.I., Chenova, O.A., et al., 1967. Keys to the insects of the European USSR. Academy of Sicences of the USSR, printed in Jerusalem
5. Beier, M., 1963. Ordnung Pseudoscorpionidea (Afterskorpione). In Bestimmungsbücher zur Bodenfauna Europas, vol. 1. Akademie-Verlag: Berlin.
6. Bolton, B., 1994. Identification Guide of the Ant Genera of the World. Harvard University Press, Cambridge, Massachusetts
7. Bonato, L., Minelli, A., Lopresti, M., Cerretti, P., 2014. ChiloKey, an interactive identification tool for the geophilomorph centipedes of Europe (Chilopoda, Geophilomorpha). ZooKeys 443: 1-9 http://www.interactive-keys.eu/chilokey/default.aspx
8. Brescovit, A.D., Rheims, C.A., Bonaldo, A.B., 2007. Chave de Identificação para Famílias de Aranhas Brasileiras, 19pp
9. Brescovit, A.D., Oliveira, U., Santos, A.J., 2011. Aranhas (Aranaea, Arachnida) do Estado de São Paulo, Brasil: diversidade, esforço amostral e estado do conhecimento. Biota Neotropica, 11(1):1-31
10. Buddle, C.M., 2010. Photographic key to the Pseudoscorpions of Canada and the adjacent USA. Canadian Journal of Arthropod Identification, 10:1-77
11. Bueno-Villgas, J., Sierwald, P., Bond, J.E., 2004. Diplopoda. Pages 569-579. https://www.academia.edu/241453/Diplopoda._Chapter_22. (accessed on 8/5/2014)
12. Chen, S., 1946. Evolution of the insect larva. Transaction of the Royal Entomological Society of London, 97:381-404
13. Chu, H.F., 1949. The Immature Insects. M C Brown Company Publishers, Dubuque Iowa
14. Crotty, F., Shepherd, M., 2014. A key to soil mites in the UK. Test Version 2. tombio.myspecies.info/SoilMitesKey# (accessed on December-2015)
15. CSRIO, 1990. The Insects of Australia, Vol. 1, Second Edition. Melbourne University Press, Carlton, Austrália
16. CSRIO, 1991. The Insects of Australia, Vol. 2, Second Edition. Melbourne University Press, Carlton, Austrália
17. CSRIO, 2009. World Thysanoptera. http://anic.ento.csiro.au/thrips/ (accessed on January-2016)
18. CSRIO, 2015. Key to Australian Freshwater and Terrestrial Invertebrates. http://keys.lucidcentral.org/keys/v3/TFI/content/Keys.html (accessed on December-2015)
19. Dioguardi, R., 2015. Chilobase: a web resource for Chiolopoda taxonomy. http://chilobase.bio.unipd.it/ (accessed on December-2015)
20. Fernández, F., 2003. Introducción a las Hormigas de la Región Neotropical. Instituto de Investigación de Recursos Biológicos Alexander von Humboldt, Bogotá
21. Foddai, D., Pereira, L.A., Minelli, A., 2004. The geophilomorph centípedes (Chilopoda) of Brazilian Amazonia. Anales del Instituto de Biología, Universidad Nacional Autónoma de México, Serie Zoología 75(2):271-282
22. Harvey, M.S., 2013. Pseudoscorpions of the World, version 3.0. Western Australian Museum, Perth. http://www.museum.wa.gov.au/catalogues/pseudoscorpions (accessed December 2015)
23. Hawkeswood, T.J., 2003. Spiders of Australia: An Introduction to their Classification, Biology and Distribution. Pensoft Publishers, Sofia, Bulgaria
24. Janssens, F., 2014. Checklist of the Collembola. http://www.collembola.org (accessed on December-2015)
25. Jordana R., Arbea, J.I., 1989. Clave de identificación de los géneros de Colémbolos de España (Insecta:Collembola). Publicaicones de Biología de la Universidad de Navarra, Serie Zoologica, 19:1-35

26. Kenneth, A.C., Greenslade, P., Deharveng, L., Pomorski, R.J., Janssens, F., 2015. Key to the families of Collembola. http://www.collembola.org/key/collembo.htm (accessed on December-2015)
27. Krantz, G.W., Walter, D.E., 2009. Acarology. Texas Tech Univesity Press, Lubbock, Texas
28. Lawrence, J.F., Hastings, A.M., Seago, A., Slipinski, A., 2010. Beetles of the World. CSIRO Entomology. http://keys.lucidcentral.org/keys/v3/botw/ (accessed on December-2015)
29. Moraes, G.J., Flechtmann, C.H.W., 2008. Manual de Acarologia: Acarologia Básica e Ácaros de Plantas Cultivadas no Brasil. Holos Editora Ltda-ME, Ribeirão Preto, São Paulo
30. Nentwig, W., Blick, T., Gloor, D., Hänggi, A., Kropf, K., 2015. Araneae: spiders of Europe. araneae Version 11. http://www.araneae.unibe.ch/key (accessed on December-2015)
31. Rafael, J.A., 2012. Insetos do Brasil: Diversidade e Taxonomia. Holos Editora Ltda-ME, Ribeirão Preto, São Paulo
32. Ramos, M.A., 2016. Fauna Ibérica. Museo Nacional de Ciencias Naturales (CSIC), Dep. de Biodiversidad y Biologia Evolutiva http://www.fauna-iberica.mncn.csic.es/# (accessed on January-2016)
33. Schuh, R.T., Slater, J.A., 1995. True bugs of the world (Hemiptera:Heteroptera). Cornell University Press, New York
34. Sierwald, P., 2015. Milli-PEET: Key to Millipede Orders. The Field Museum, Chicago, USA. https://www.fieldmuseum.org/science/special-projects/milli-peet-class-diplopoda/milli-peet-millipedes-made-easy/milli-peet-key (accessed on December-2015).
35. Smith, K.G.V., 1989. An Introduction to the Immature Stages of British Flies: Diptera Larvae, with Notes on Eggs, Puparia and Pupae. Royal Entomological Society of London, London
36. Smithers, C.N., 1967). A catalogue of the Psocoptera of the world. The Australian Zoologist 14(1): 1-145
37. Smithers, C.N., 1990. Keys to the families and genera of Psocoptera (Arthropoda: Insecta). Technical Reports of the Australian Museum, 2:1-82.
38. Stehr, F.W., 1987. Immature Insects, Volume 1. Kendall-Hunt, Dubuque, Iowa
39. Triplehorn, C.A., Johnson, N.F., 2005. Borror and DeLong's lntroduction to the Study of lnsects. Thomson Brooks/Cole, USA
40. Thyssen, P.J., 2010. Keys for Identification of Immature Insects. Pages 25-42 In: Amendt J, Goff ML, Campobasso CP, Grassberber, M (Eds). Current Concepts in Forensic Entomology. Springer
41. Walter, D.E., 2011. Families of Parasitiformes in soil. version 1.0. The University of Queensland. http://keys.lucidcentral.org/ (assessed in December-2015)
42. Walter, D.E., Proctor, H.C., 2011. Soil Microarthropods. Version 1.0. The University of Queensland. http://keys.lucidcentral.org/ (assessed in December-2015)
43. Walter, D.E., Proctor, H., 2010. Orders, Suborders & Cohorts of Mites in Soil. Version 1.0. http://keys.lucidcentral.org/ (assessed on December-2015)
44. World Spider Catalog, 2015. Natural History Museum Bern, online at http://wsc.nmbe.ch, version 15.5 (accessed on December-2015)
45. Workman, T., 1896. Spiders. Belfast Natural History and Philosophical Society, Belfast, Pensilvania
46. Zaragoza, J.A., 2006. Catálogo de los Pseudoescorpiones de la Península Ibérica e Islas Baleares (Arachnida: Pseudoscorpiones). Revista Ibérica de Aracnología, 13(30)-VI-91p
47. Zaragoza, J.A., 2001. Minniza iberica n.sp., primera cita del género para la Península Ibérica y redescripción de Minniza algerica Beier, 1931 (Arachnida, Pseudoscorpionida, Olpiidae). Revista Ibérica de Aracnología, 3: 69-78.
48. Zaragoza, J.A., 2004. Pseudoescorpiones. *In* Barrientos, J. A. Ed: Curso practico de entomologia. Asociacion Española de Entomología; Alicante: CIBIO. Centro Iberoamericano de la Biodiversidad; Bellaterra: Universitat Autònoma de Barcelona, Servei de Publicacions: 177-187.
